# Natural medicines for treating liver fibrosis by modulating post-translational modifications

**DOI:** 10.3389/fphar.2025.1660908

**Published:** 2025-11-10

**Authors:** Qun Niu, Yu Mou, Kaixin Wang, Haijian Dong, Zijian Zeng, Hui Li

**Affiliations:** 1 Hospital of Chengdu University of Traditional Chinese Medicine, Chengdu, China; 2 Chengdu University of Traditional Chinese Medicine, Chengdu, China

**Keywords:** hepatic fibrosis, post-translational modifications, natural products, hepatic stellate cells, extracellular matrix, TGF-β, antifibrotic therapy

## Abstract

Hepatic fibrosis is a multifactorial process driven by hepatic stellate cell (HSCs) activation, participation of Kupffer cells and infiltrating immune cells, and profibrotic cytokine signaling (notably TGF-β), culminating in excessive extracellular matrix (ECM) and collagen deposition. Post-translational modifications (PTMs)—covalent changes added after protein synthesis—govern protein stability, localization, interactions, and activity. Common PTMs include phosphorylation, acetylation, ubiquitination, glycosylation, nitration, and methylation; collectively, they modulate fibrogenic pathways across disease stages. Despite available therapies, clinically effective and well-tolerated antifibrotic options remain limited. Natural products, with their structural diversity, relative safety, and broad accessibility, offer promising leads for antifibrotic drug discovery. This review delineates the central roles of PTMs in hepatic fibrosis, synthesizes how specific PTMs drive disease initiation and progression, and evaluates natural products that target PTM-regulated nodes of fibrogenesis. We also propose strategies to accelerate development of PTM-informed antifibrotic therapeutics.

## 1 Introduction

Hepatic fibrosis (HF) is a progressive pathological condition that substantially contributes to the global disease burden because it can advance to cirrhosis, liver failure, and hepatocellular carcinoma—each associated with high morbidity and mortality (Huang et al., 2023). Pathologically, HF features excessive deposition of collagen and other extracellular matrix (ECM) components that, while central to normal wound healing, become dysregulated in persistent injury and inflammation (Friedman, 2008; Huang et al., 2020; Seki and Schwabe, 2015). Such fibrotic remodeling impairs organ function and underlies major complications, including cirrhosis, renal failure, and myocardial fibrosis–related heart failure (McQuitty et al., 2020). In the liver, fibrosis represents a common, dynamic response across chronic liver diseases (CLDs) and is a key determinant of progression to cirrhosis, hepatocellular carcinoma, and ultimately liver failure (Zhou et al., 2020). Its pathogenesis reflects complex crosstalk among hepatocytes, hepatic stellate cells (HSCs), sinusoidal endothelial cells, and resident and infiltrating immune cells (Kisseleva and Brenner, 2021; Tsuchida and Friedman, 2017). Following acute injury, hepatocytes typically regenerate and replace necrotic or apoptotic cells to restore tissue integrity (Michalopoulos, 2017; Michalopoulos and Bhushan, 2021); with chronic injury, however, this regenerative capacity progressively fails, and parenchyma is increasingly replaced by ECM (Baiocchini et al., 2016; Ginès et al., 2021; Tsuchida and Friedman, 2017).

During fibrogenesis, virtually all hepatic cell types—parenchymal and non-parenchymal—undergo characteristic alterations (Seo and Jeong, 2016). Injured hepatocytes undergo apoptosis, whereas liver sinusoidal endothelial cells lose their fenestrations, resulting in sinusoidal capillarization (Canbay et al., 2004). Liver injury also activates Kupffer cells, the resident macrophages, which release cytokines and chemokines (Dixon et al., 2013; van der Heide et al., 2019). These mediators drive the transition of quiescent HSCs into an activated, myofibroblast-like state marked by *de novo* expression of platelet-derived growth factor (PDGF) receptors, transforming growth factor-β (TGF-β) receptors, and α-smooth muscle actin (α-SMA). Activated HSCs proliferate and secrete ECM components, ultimately depositing fibrotic scar tissue (Dewidar et al., 2019; Marcher et al., 2019; Ying et al., 2017).

**FIGURE 1:**
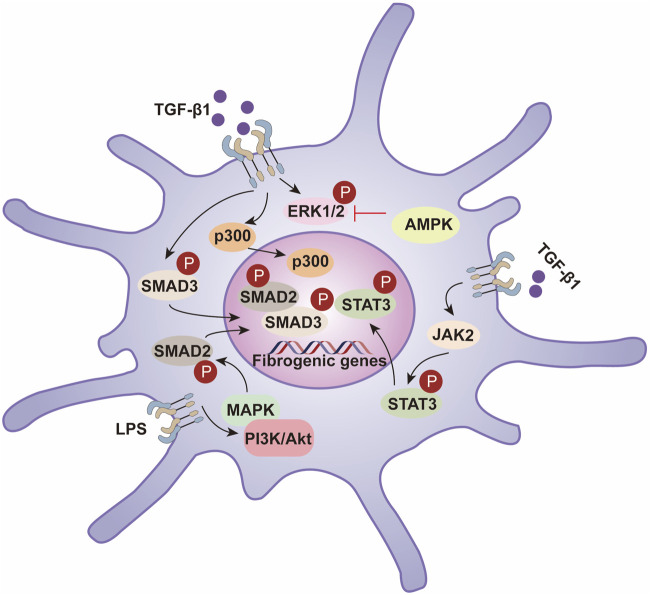
Hepatic fibrosis progresses through hepatic stellate cell activation, leading to ECM deposition and fibrotic scarring. PTM such as phosphorylation, glycosylation, and ubiquitination regulate protein function and play critical roles in fibrosis pathogenesis.

The progression of liver fibrosis is driven by diverse cellular programs and regulatory networks. Key molecular effectors in hepatic fibrogenesis include TGF-β receptors, SMAD transcription factors, and extracellular-matrix–modifying enzymes, which drive stellate-cell activation and matrix remodeling. Beyond synthesis, PTMs introduce specific chemical changes that reshape protein activity, stability, localization, and interactions, adding a crucial regulatory layer to the pathological remodeling of the liver. While gene transcription and translation establish the proteome, PTMs dynamically define protein function in context. Accordingly, the addition, removal, or rearrangement of functional groups can markedly alter protein behavior and thereby influence disease initiation and progression (Shu et al., 2023) (Figure 1).

More than 400 PTM types have been reported to date; among the most prevalent are phosphorylation, acetylation, ubiquitination, glycosylation, nitration, and methylation (Wu and Jankowski, 2022). Nevertheless, comprehensive mapping of PTM substrates and elucidation of their functional consequences—especially in liver disease—remain incomplete. This review synthesizes current advances on PTMs in hepatic fibrosis, outlines key knowledge gaps, and discusses how PTM-focused insights may inform novel therapeutic targets and drug development.

## 2 Literature screening and selection process

To comprehensively review the role of post-translational modifications (PTMs) in liver fibrosis and the regulatory effects of natural products on PTMs, a structured literature search was performed in accordance with the PRISMA 2020 guidelines.

### 2.1 Databases and time frame

Electronic databases including PubMed/MEDLINE, Web of Science Core Collection, Embase, and Scopus were searched for publications from January 2000 to June 2024. Additional references were retrieved by manually screening the bibliographies of relevant reviews and primary articles.

### 2.2 Search strategy

A combination of Medical Subject Headings (MeSH) and free-text terms was used to capture three major concepts:

Disease: “liver fibrosis” OR “hepatic fibrosis” OR “hepatic stellate cell*” OR “liver cirrhosis”.

Modification: “post-translational modification” OR “PTM” OR “phosphorylation” OR “acetylation” OR “ubiquitination” OR “SUMOylation” OR “methylation” OR “succinylation” OR “malonylation” OR “glycosylation”.

Natural products: natural product” OR “phytochemical” OR “herbal compound*” OR “traditional medicine” OR names of key classes (“flavonoid” OR “saponin” OR “alkaloid” OR “polyphenol”).

The three groups were combined using AND (e.g., liver fibrosis AND post-translational modification AND natural product). Searches were adapted for the syntax of each database.

### 2.3 Eligibility criteria

Inclusion: i. original experimental or clinical studies evaluating any PTM in the context of liver fibrosis and reporting modulation by a natural product or plant-derived compound; ii. *in vitro* (e.g., hepatic stellate cell activation), *in vivo* (animal models), or human clinical studies; iii. Articles in English with full text available.

Exclusion: reviews, editorials, conference abstracts without primary data; studies on liver cancer or metabolic liver disease lacking fibrosis/PTM endpoints; reports of genetic polymorphisms without PTM assessment.

### 2.4 Screening procedure

Two independent reviewers screened titles and abstracts for relevance. Full texts of potentially eligible studies were retrieved for detailed assessment. Disagreements were resolved through discussion or consultation with a third reviewer.

### 2.5 Data extraction and quality assessment

For each included study, we extracted: Type of natural product or compound (e.g., flavonoid, saponin, alkaloid); PTM type (phosphorylation, acetylation, ubiquitination, SUMOylation, methylation, succinylation, malonylation, etc.); Target proteins/enzymes (writers, erasers, readers) and signaling pathways.

## 3 Protein post-translational modifications in liver fibrosis

### 3.1 Phosphorylation modifications

Protein phosphorylation is a ubiquitous, deeply studied PTM that governs fundamental biological programs—including cell growth, differentiation, apoptosis, gene expression, and signal transduction (Bilbrough et al., 2022). In liver fibrosis, phosphorylation is tightly coupled to disease initiation and progression, largely through effects on ECM deposition and HSCs activation (Okuno et al., 2001). HSCs release and activate transforming growth factor-β1 (TGF-β1) and its intracellular Smad mediators, both of which are pivotal for driving collagen gene transcription (Inagaki et al., 2005; Okuno et al., 2001). TGF-β ligands also promote ECM accumulation and become sequestered within the matrix, thereby amplifying profibrotic signaling (Walton et al., 2017). Following injury, elevated TGF-β in the fibrotic niche activates SMAD2/3 via TGF-β receptors 1/2 (TGFBR1/2) (Budi et al., 2021). The phosphorylated SMAD2/3 complex then translocates to the nucleus to directly induce downstream targets (e.g., COL1A1, COL3A1, COL5A2), initiating ECM gene expression and advancing fibrogenesis (Walton et al., 2017).

Additional upstream cues converge on Smad signaling through canonical kinase cascades. For example, lipopolysaccharide (LPS) induces SMAD2 phosphorylation in HSC-T6 cells via PI3K/AKT and MAPK pathways, promoting ECM production and myofibroblastic transition (Kao et al., 2017). Renin/prorenin signaling increases prorenin receptor (PRR) expression and TGF-β1 production in LX-2 cells; PRR knockdown deactivates HSCs, reduces TGF-β1, and diminishes SMAD3 phosphorylation, thereby alleviating fibrotic responses (Hsieh et al., 2021).

Phosphorylation also regulates hepatocyte fate programs relevant to fibrosis. During hepatocyte apoptosis, caspase activity is modulated by phosphorylation—phospho-caspase-9, for instance, promotes apoptosis—typically accompanied by heightened inflammation and secondary HSC activation (Cardone et al., 1998; Cui et al., 2020; Riedl and Salvesen, 2007). Autophagy, another phosphorylation-tuned process, fuels HSC activation by mobilizing energy from retinoid-rich lipid droplets; its pharmacologic inhibition suppresses HSC activation and mitigates fibrosis *in vitro* and *in vivo* (Hernandez-Gea et al., 2012; Thoen et al., 2011). Mechanistically, Sestrin2 may restrain HSC activation by enhancing AMPK phosphorylation and dampening mTOR signaling (Hu Y. J. et al., 2024), whereas the BET inhibitor JQ-1 improves fibrosis by limiting HSC activation and proliferation through reduced JAK2/STAT3 phosphorylation (Song et al., 2023a).

**FIGURE 2:**
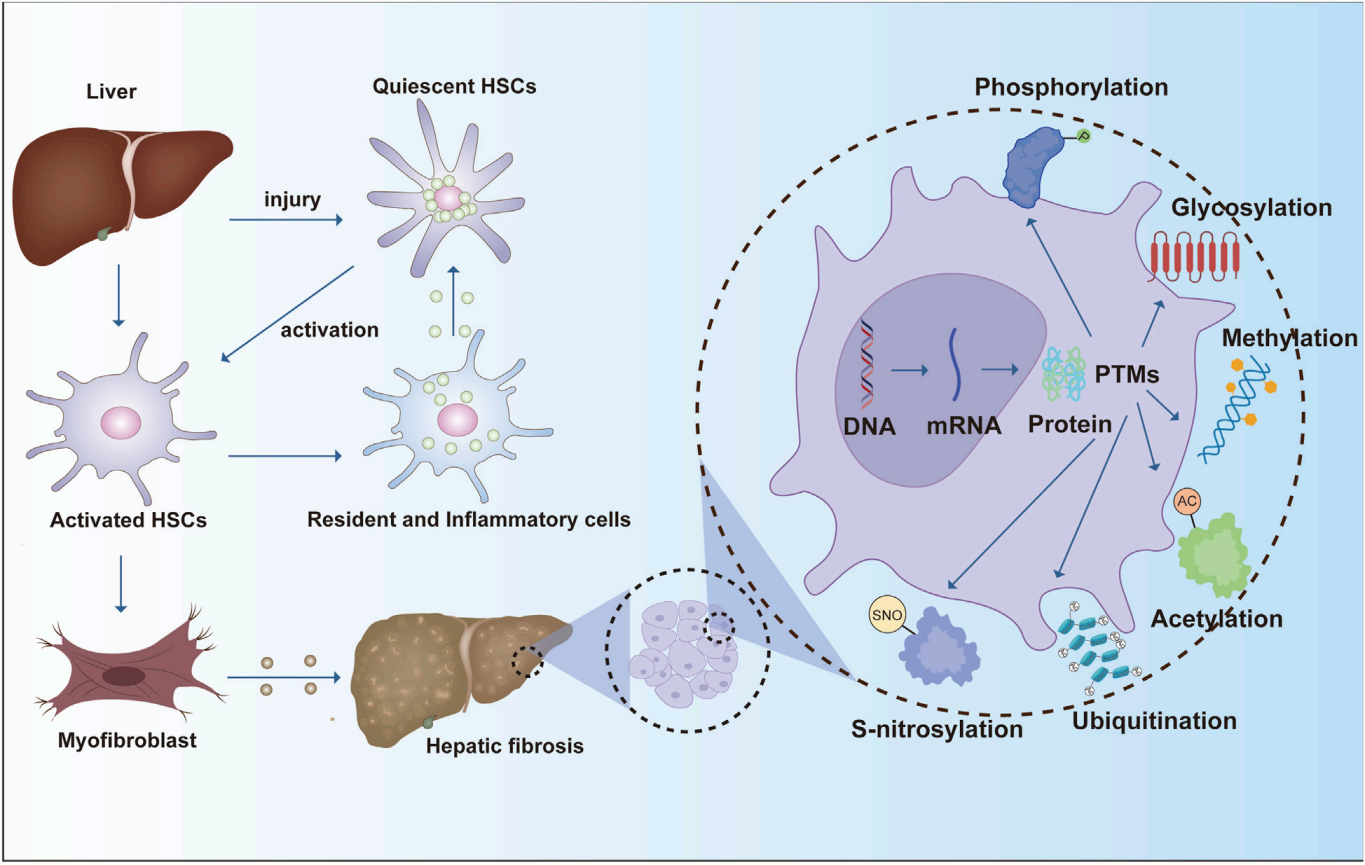
Hepatic stellate cell activation is driven by phosphorylation-dependent signaling pathways that regulate fibrogenesis. TGF-β1 activates the SMAD2/3 pathway, promoting fibrogenic gene expression through SMAD3 phosphorylation and interaction with p300. STAT3 activation via JAK2 and PI3K/Akt pathways enhances fibrosis, while LPS-induced MAPK signaling further contributes to this process. AMPK inhibits fibrosis by suppressing ERK1/2 phosphorylation, balancing profibrotic and antifibrotic mechanisms in liver fibrosis.

In sum, phosphorylation exerts broad, stage-spanning control over fibrogenesis by modulating key signaling axes (TGF-β/Smad, MAPK, NF-κB, PI3K/AKT) and by shaping HSC activation, proliferation, apoptosis, and autophagy (Dooley and ten Dijke, 2012; Hernandez-Gea and Friedman, 2011). Deeper delineation of these phosphorylation-dependent mechanisms—and their node-specific roles—will aid target identification and inform the rational development of antifibrotic therapies (Figure 2).

### 3.2 Protein glycosylation modification

Altered protein glycosylation is a key driver of hepatic fibrogenesis (Eichler, 2019). Glycosylation occurs co- and post-translationally as nascent proteins enter the endoplasmic reticulum, where enzymes install monosaccharides that are subsequently elaborated into oligosaccharide chains in a site-specific manner (Hebert et al., 2005). Two major classes predominate: N-glycosylation on the amide nitrogen of asparagine (Asn) and O-glycosylation on the hydroxyl groups of serine/threonine (Ser/Thr) (Kornfeld, 1998). In liver fibrosis, glycosylation shapes both matrix composition and profibrotic signaling. For example, glycan-dependent Galectin-1/NRP1 interactions potentiate TGF-β and PDGF-like pathways to activate HSCs, while the collagen O-galactosyltransferase GLT25D1 enhances collagen O-glycosylation and stabilizes fibrotic matrix architecture (Wang S. et al., 2023; Wu et al., 2017).

Glycosylation participates directly in ECM deposition by modifying collagens and thereby influencing their stability and susceptibility to degradation. It also regulates HSC activation (Caon et al., 2021; Ogawa and Okajima, 2019; Rockey et al., 2015). A particularly important cytosolic modification is O-GlcNAcylation, which decorates nuclear, cytoplasmic, and mitochondrial proteins. O-GlcNAc transferase (OGT) installs, and O-GlcNAcase (OGA) removes, this modification (Hahne et al., 2013; Ma and Hart, 2014; Ruan et al., 2012). In myofibroblast-like HSCs (MF-HSCs), global O-GlcNAcylation rises in parallel with α-SMA expression; similar increases occur in CCl_4_-induced liver injury in mice. Pharmacologic OGT inhibition (e.g., OSMI-1) lowers O-GlcNAcylation and downregulates collagen genes (Col1a1, Col1a2, Col3a1, Col5a2), indicating that O-GlcNAcylation is required for robust expression of fibrosis-related ECM genes and is a determinant of myofibroblast activation (Harvey and Chan, 2018; Housley et al., 2009; Wang et al., 2022).

Glycan-based biomarkers also mirror disease activity. Mac-2 binding protein glycoforms (M2BPGi)—a glycosylated variant of M2BP produced predominantly by HSCs—serve as serum indicators of liver fibrosis. M2BPGi engages Mac-2 on Kupffer cells, which in turn promotes HSC activation and elevates α-SMA expression (Bekki et al., 2017; Gantumur et al., 2021).

Glycosylation intersects with oxidative stress through advanced glycation end products (AGEs), non-enzymatic adducts formed between reducing sugars and proteins, lipids, or nucleic acids. AGE accumulation augments oxidative stress and activates pro-fibrotic, pro-inflammatory signaling via the receptor for AGEs (RAGE) (Hollenbach, 2017; Hyogo and Yamagishi, 2008). *In vitro*, glyceraldehyde-derived AGEs increase ROS, induce chronic injury signals, and drive HSC activation (Iwamoto et al., 2008). Consistently, RAGE expression is upregulated during HSC transdifferentiation to myofibroblasts, reinforcing profibrotic pathways (Fehrenbach et al., 2001).

**FIGURE 3:**
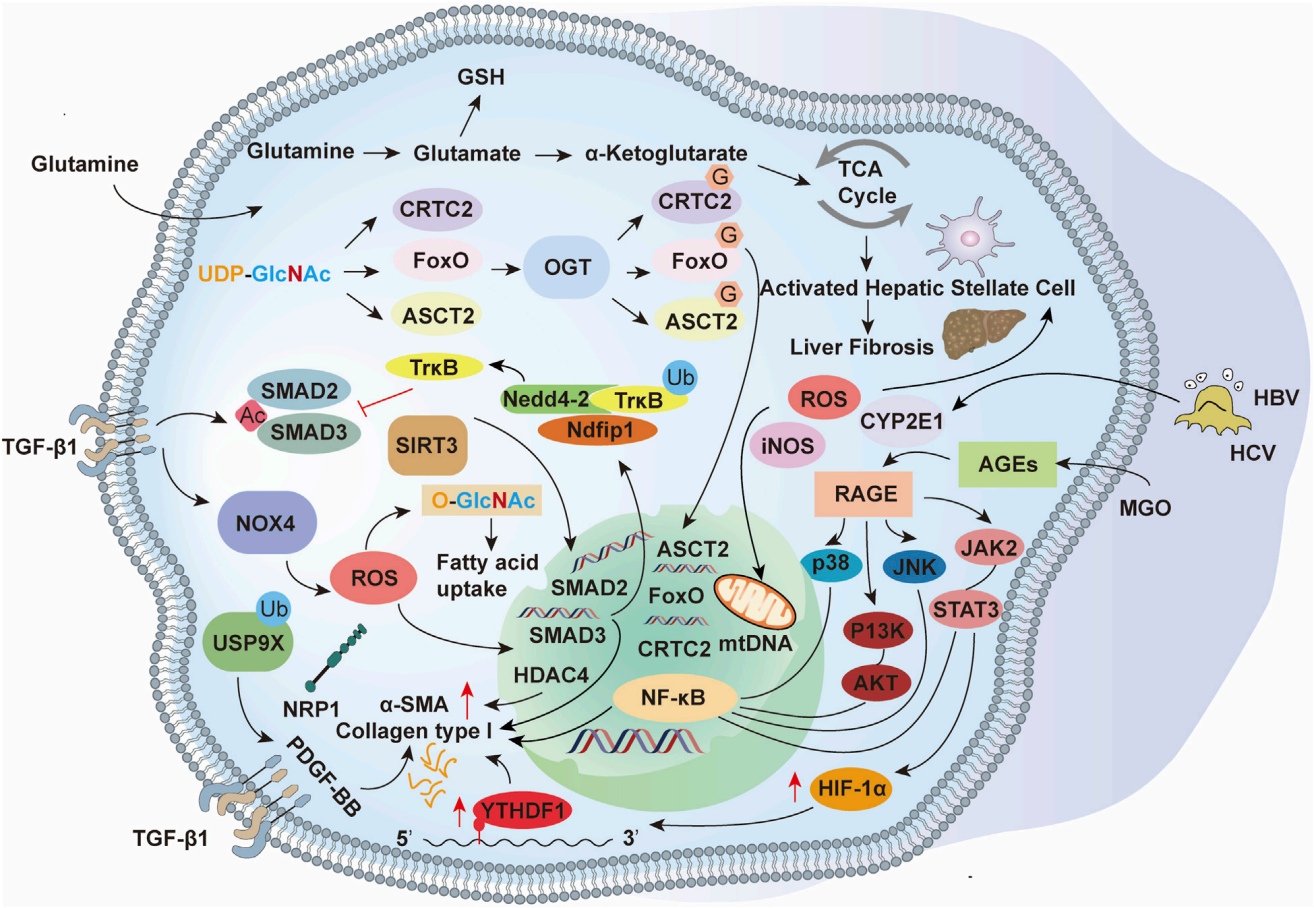
The activation of HSCs during liver fibrosis is orchestrated by PTMs, which regulate key signaling pathways and cellular processes. Acetylation modulates chromatin accessibility and transcriptional activity, ubiquitination governs protein turnover and degradation, and glycosylation influences protein folding, stability, and intercellular signaling. These modifications, in conjunction with TGF-β1-induced SMAD2/3 activation, oxidative stress, and metabolic reprogramming, drive HSC activation, extracellular matrix production, and fibrotic remodeling.

Collectively, these findings position glycosylation—enzymatic glycans and non-enzymatic AGEs alike—as a central regulatory layer in hepatic fibrogenesis. Targeting specific glycosylation enzymes, lectin-mediated interactions, or AGE–RAGE signaling may yield promising antifibrotic strategies (Figure 3).

### 3.3 Protein acetylation modification

Protein acetylation—installed by acetyltransferases and removed by deacetylases—adds acetyl groups to specific residues, reshaping chromatin architecture, transcription, and signal transduction, and thereby influencing gene expression, protein function, cellular metabolism, and cell-cycle progression (Arnesen, 2011; Verdin and Ott, 2015; Xue et al., 2022). In hepatic fibrosis, epigenetic dysregulation driven by histone modifications is a major determinant of disease progression (Tessarz and Kouzarides, 2014; Tsukamoto et al., 2011). Activation and transdifferentiation of HSCs require broad epigenetic reprogramming that silences adipogenic programs while inducing genes supporting the myofibroblast-like phenotype (Barcena-Varela et al., 2019; Mann and Mann, 2013; Moran-Salvador and Mann, 2017).

A growing body of evidence implicates acetylation in HSC activation and fibrogenesis. Acetylation of C/EBP-α at K298, K302, and K326 enhances its interaction with Beclin-1 and promotes autophagy in activated HSCs (Hou et al., 2021). Pharmacologic inhibition of histone deacetylases with trichostatin A (TSA) alleviates CCl_4_-induced fibrosis, in part by increasing C/EBP-α acetylation and blocking its ubiquitin-dependent degradation (Ding et al., 2018). More broadly, hyperacetylation at promoters/enhancers of profibrotic genes associates with elevated TGF-β, collagen, and α-SMA expression. Acetylation also augments NF-κB activity, increasing TNF-α and IL-6 production that further activates HSCs; conversely, HDAC-mediated deacetylation dampens NF-κB signaling and mitigates inflammatory drive and fibrogenesis (Jimenez-Uribe et al., 2021; Joanna et al., 2009; Park et al., 2014). In LX-2 cells, nicotinamide riboside (NR) restrains TGF-β–induced activation by modulating acetylation within the Smad pathway and reduces CCl_4_-induced fibrosis *in vivo* (Jiang et al., 2019).

Acetylation also directly tunes mitochondrial function, influencing cellular energetics and oxidative stress (Anderson and Hirschey, 2012). The mitochondrial deacetylase SIRT3 deacetylates key antioxidant enzymes, attenuating oxidative stress and slowing fibrotic progression (Ning et al., 2024). Additionally, acetylation of apoptosis regulators within the Bcl-2 family can shift cell-death propensity. Some studies suggest that acetylation may increase the activity of pro-apoptotic proteins, promoting the apoptosis of HSCs and limiting the extent of fibrosis (Ma et al., 2022; Zhang et al., 2022). Together, these findings position acetylation—as both a chromatin-level and protein-level switch—as a tractable axis for antifibrotic intervention (Figure 3).

### 3.4 Protein ubiquitination modification

Ubiquitination is a covalent modification in which a small ubiquitin protein is conjugated to target proteins through a cascade of enzymatic reactions (Dikic and Schulman, 2023). This process plays a central role in protein degradation, signal transduction, cell cycle control, DNA repair, immune responses, and other cellular activities (Popovic et al., 2014). It requires the coordinated action of three enzymes: the E1 ubiquitin-activating enzyme, which activates ubiquitin; the E2 ubiquitin-conjugating enzyme, which transfers activated ubiquitin; and the E3 ubiquitin ligase, which attaches ubiquitin to lysine residues of substrate proteins, thereby conferring specificity (Pickart, 2001). Ubiquitination can occur as monoubiquitination (attachment of a single ubiquitin molecule) or polyubiquitination (formation of ubiquitin chains), with the type of linkage determining the fate of the substrate protein (Nakamura, 2018).

Accumulating evidence highlights the pivotal role of ubiquitination in liver fibrosis, regulating signaling pathways, protein turnover, cell activation, and programmed cell death (Filali-Mouncef et al., 2022; Rao et al., 2022; Shu B. et al., 2021; Wu et al., 2023). Ubiquitin has been identified as a biomarker of nonalcoholic liver fibrosis, frequently detected at cell boundaries and within the fibrotic matrix (Lachiondo-Ortega et al., 2019). The deubiquitinating enzyme UCHL1 is markedly upregulated in human HSCs and in livers of patients with alcoholic liver disease. Pharmacologic inhibition of UCHL1 with LDN-57444 mitigates fibrosis progression in CCl_4_-induced mouse models (Wilson et al., 2015). Similarly, the E3 ligase FBG1 degrades misfolded A1AT-Z mutants through the ubiquitin–proteasome system and autophagy, preventing their accumulation in the endoplasmic reticulum and attenuating fibrogenic stress (Wen et al., 2015).

Other ubiquitin-related mechanisms also contribute to fibrogenesis. Hepatocyte-specific deletion of the E3 ligase RNF5 exacerbates steatosis, inflammation, and fibrosis in dietary NASH models. Mechanistically, RNF5 binds HRD1 and promotes its K48/K33-linked ubiquitination, leading to proteasomal degradation; HRD1 knockdown reduces lipid accumulation and inflammatory signaling, underscoring the RNF5–HRD1 axis in hepatocyte injury and fibrosis (Yang et al., 2021). In HSCs, TGF-β signaling induces Ndfip1 expression, which recruits the E3 ligase Nedd4-2 to promote ubiquitination and degradation of TrkB. TrkB overexpression suppresses TGF-β/Smad signaling and limits HSC proliferation, suggesting that TrkB ubiquitination contributes to fibrosis progression (Song et al., 2023b). Moreover, Neuropilin-1 (NRP1) enhances HSC activation via TGF-β1, VEGFA, and PDGF-BB, while the deubiquitinase USP9X stabilizes NRP1. Thus, USP9X-mediated deubiquitination amplifies HSC activation, making the USP9X–NRP1 axis a promising therapeutic target (Zhao et al., 2023b).

Ubiquitination also intersects with hepatocyte injury and apoptosis. The transcription factor NRF2, a key antioxidant regulator, is degraded via ubiquitination by the CUL3–KEAP1 E3 ligase complex, a process dependent on neddylation (Zhang et al., 2004). Impaired neddylation disrupts NRF2 stability, induces mitochondrial dysfunction, and exacerbates oxidative stress, thereby promoting hepatocyte death and fibrosis (Xu et al., 2022). In addition, TRAF6 mediates K6-linked ubiquitination of apoptosis signal-regulating kinase 1 (ASK1), facilitating dissociation from thioredoxin and promoting ASK1 dimerization. This event activates the ASK1–JNK1/2–p38 cascade, stimulating pro-inflammatory and pro-fibrotic mediators that drive HSC activation and fibrogenesis (Wang et al., 2020b).

In summary, ubiquitination exerts multifaceted regulatory control over liver fibrosis by modulating HSC activation, ECM metabolism, inflammation, and apoptosis. Elucidating the precise ubiquitin-dependent mechanisms in fibrogenesis may reveal novel molecular targets and foster the development of innovative antifibrotic therapies (Figure 3).

### 3.5 Protein nitration modification

Protein S-nitrosylation is a covalent NO-dependent modification that regulates cell signaling by altering protein–protein interactions, subcellular localization, stability, and reactivity (Zhao et al., 2021). In parallel, protein nitration—most commonly tyrosine nitration mediated by peroxynitrite formed from NO and superoxide—affects transcriptional control, DNA-damage responses, cell growth, differentiation, and apoptosis; dysregulation of these NO-linked processes contributes to diverse diseases (Fernando et al., 2019).

Extensive evidence implicates NO-mediated modifications in hepatic fibrogenesis. Svegliati-Baroni et al. showed that the exogenous NO donor S-nitroso-N-acetylpenicillamine (SNAP) suppresses HSC activation and proliferation by scavenging ROS, thereby limiting the onset of hepatic fibrosis and cirrhosis (Svegliati-Baroni et al., 2001). Within the space of Disse, liver sinusoidal endothelial cells (LSECs) release VEGF, which supports HSC proliferation and angiogenesis; under physiological conditions, VEGF-stimulated eNOS activity in LSECs generates NO that helps revert activated HSCs toward quiescence (Deleve et al., 2008). Consistently, eNOS-derived NO is generally protective, whereas iNOS-derived NO is linked to pathological nitrosative stress and disease progression (Iwakiri and Kim, 2015).

Nitrosative stress is elevated in obesity-related NASH and in models of chronic fructose exposure, as evidenced by increased CYP2E1, iNOS, and protein nitration, changes that associate with fibrotic remodeling (Cho et al., 2021). In HepG2 cells replicating HBV or expressing HBx, mitochondrial superoxide and peroxynitrite rise, leading to mtDNA damage, nitration of respiratory-chain complexes—especially complex I—and bioenergetic impairment; superoxide scavenging (Mito-Tempo) or iNOS inhibition prevents these lesions, implicating mitochondrial nitrosative damage in inflammation and profibrotic signaling (Loureiro et al., 2023). Heat-shock protein 90 (HSP90), highly expressed in hepatocytes, is a nitration target: in aged wild-type mice, HSP90 nitration accompanies oxidative DNA damage, increased mitochondrial nitrosative stress, and alterations in complexes III/IV, culminating in age-dependent steatosis, apoptosis, and fibrosis (Abdelmegeed et al., 2016).

Given the central role of NO-driven S-nitrosylation and nitration in liver fibrosis, therapeutic strategies that rebalance these pathways are promising: enhancing eNOS-derived cytoprotective NO, limiting iNOS-driven nitrosative stress, curbing peroxynitrite formation, and/or strengthening antioxidant defenses may attenuate fibrogenic progression (Figure 3).

### 3.6 Methylation modification

Methylation is a major post-translational modification occurring predominantly on lysine and arginine residues of histone and non-histone proteins. By shaping chromatin architecture, gene expression, and intracellular signaling, it exerts wide-ranging control over cellular phenotypes; its dysregulation is closely linked hepatic HSCs activation and liver fibrosis (Mattei et al., 2022; Menezo et al., 2020).

Emerging evidence indicates that the methylation status of fibrotic regulators serves not only as a biomarker but also as a functional driver of disease progression (Yang et al., 2020). For example, PTEN antagonizes PI3K lipid signaling and thereby restrains PI3K/AKT and ERK pathways implicated in HSC activation (Kumar et al., 2018). In TGF-β–stimulated HSCs, the DNA methylation inhibitor 5-Aza sustains PTEN expression, attenuating HSC activation and alleviating fibrosis (Bian et al., 2013). In a CCl_4_ model, osteopontin (Spp1) is markedly upregulated; hypomethylation of the Spp1 promoter enhances its transcription, activates PI3K/AKT, promotes profibrogenic mediators (including TGF-β), and increases type I collagen and α-SMA, thereby accelerating HSC activation and matrix remodeling. Persistent activation of this axis has also been implicated in the transition from fibrosis to hepatocarcinogenesis (Song et al., 2019).

DNA methyltransferases (DNMTs) further integrate methylation cues into fibrogenic programs. DNMT1—the maintenance methyltransferase—is elevated in human cirrhotic livers, murine fibrosis, and primary mouse HSCs; in human HSCs, TGF-β1 recruits DNMT1 to chromatin. Genetic DNMT1 knockdown or pharmacologic disruption of the G9a/DNMT1 complex with CM272 suppresses TGF-β1–driven fibrotic responses and mitigates fibrosis (Barcena-Varela et al., 2021). DNMT3b also contributes by repressing SUN2: during CCl_4_-induced fibrosis, CpG hypermethylation coincides with low SUN2 expression. AAV9-mediated SUN2 overexpression reduces fibrotic markers *in vivo*, and SUN2 overexpression in TGF-β1–activated HSC-T6 cells dampens HSC activation (Chen et al., 2018).

Beyond DNA, RNA methylation intersects with fibrogenesis. Coordinated waves of 5-methylcytosine (5 mC, DNA) and N6-methyladenosine (m6A, RNA) modifications align with distinct phases of HSC activation (Luo H. et al., 2023). During initiation, promoter 5 mC hypermethylation at SOCS3 and PPARγ facilitates STAT3-dependent metabolic reprogramming and lipid loss. During maintenance, m6A hypermethylation of collagen transcripts enhances mRNA stability via YTHDF1, driving excessive ECM production (Feng et al., 2023).

The dynamic—and reversible—nature of methylation makes it an attractive therapeutic axis. Modulating methyltransferases/demethylases can reset profibrotic programs: DNA demethylating agents or histone demethylase inhibitors have been shown to restore more physiological methylation states at key loci (e.g., TGF-β1), reduce overexpression of profibrotic genes, and ameliorate fibrosis in animal models. Collectively, these findings position protein/DNA/RNA methylation as a convergent regulatory layer in hepatic fibrogenesis and a promising target space for antifibrotic drug development (Figure 3).

## 4 Natural products targeting PTMs in liver fibrosis

Natural products have long served as a valuable reservoir for drug discovery and development. Flavonoids, phenolics, terpenoids, polysaccharides, and alkaloids derived from plants exhibit diverse pharmacological activities, including anti-inflammatory, antioxidant, apoptosis-regulating, and antifibrotic effects. Importantly, many of these compounds exert their therapeutic benefits by modulating post-translational modifications (PTMs) (Table 1).

**TABLE 1:** Post-translational modifications of key compounds in liver fibrosis treatment.

Natural products	Metabolite	Source	Mechanisms	Mode of action	Model	Dosages	References
Flavonoids	Total flavonoids from Scabiosa comosa	*Scabiosa comosa Fisch. ex Roem. and Schult. [Caprifoliaceae]*	Phosphorylation	↓ p-Smad3; ↓ Smad3-TβRI interaction	In a rat model of hepatic fibrosis induced by CCl_4_; Primary mouse HSCs	50,100, and 200 mg/kg for 7 days; 25, 50 and 100 µg/mL for 1 h	Ma et al. (2018)
	Luteolin	*Limnophila aromatica (Lam.) Merr. [Plantaginaceae]*	Phosphorylation	↓ AKT/mTOR/p70S6K signalling pathways; ↓ TGFβ/Smad signaling pathways	In mice model of hepatic fibrosis induced by CCl_4_, DMN or BDL; HSC-T6 cells treated with TGF-β1	150 mg/kg for 12 weeks in CCl4 model, 150 mg/kg for 2 weeks in DMN and BDL model; 10, 20 and 40 µg/mL for 24 h	Li et al. (2015)
	Naringin	*Citrus × aurantium f. aurantium [Rutaceae]*	Phosphorylation	↓ PI3K/Akt signaling pathway	In a rat model of hepatic fibrosis induced by TAA	40 mg/kg for 6 weeks	El-Mihi et al. (2017)
	Myricetin	*Myrica rubra (Lour.) Siebold and Zucc. [Myricaceae]*	Phosphorylation	↓ p-Smad2, ↓ p-AKT, ↓ p-ERK and ↓ p-P38 MAPK	In a mice model of hepatic fibrosis induced by CCl_4;_ CFSC-8B Cells treated with TGF-β1 or PDGF-BB	50 mg/kg for 2 weeks; 12, 25 and 50 µg/mL for 2 h	Geng et al. (2017)
	Quercetin	*Houttuynia cordata Thunb. [Saururaceae]*	Phosphorylation	↓ NF-κB and p38 MAPK signaling pathways; ↓ Bcl-2/Bax anti-apoptosis signaling pathway	In a rat model of hepatic fibrosis induced by CCl_4_	5 and 15 mg/kg for 8 weeks	Wang et al. (2017)
	Limonin	*Citrus × aurantium f. aurantium [Rutaceae]*	Phosphorylation	↑ p-Smad7; ↓ p-Smad2/3	In a rat model of hepatic fibrosis induced by CCl_4_; AML-12 cell and LX-2 HSCs cell treated with TGF-β	5 and 15 μM for 24 h; 10 and 20 mg/kg for 4 weeks	Shu et al. (2022)
	Isoliquiritigenin	*Glycyrrhiza uralensis Fisch. ex DC. [Fabaceae]*	Phosphorylation	↓ p-STAT3; ↓ ANXA2 and SPHKs/S1P/IL-17 signals pathway	In a mice model of alcoholic liver fibrosis induced by alcohol feeding combined with 5% CCl_4_; HSC-T6 cells treated with alcohol	10 and 20 mg/kg for 10 days; 4, 8 and 16 μmol/L for 24 h	Liu et al. (2023)
	(−)-Catechin-7-O-β-d-apiofuranoside	*Ulmus davidiana* var. *japonica (Rehder) Nakai [Ulmaceae]*	Phosphorylation	↓ p-STAT3	In a rat model of hepatic fibrosis induced by TAA LX-2 cells treated with TGF-β1	40 mg/kg for 3 weeks; 2.5, 5, 10 and 20 μg/mL for 48 h	Park et al. (2019)
	Luteolin-7-diglucuronide	*Perilla frutescens (L.) Britton [Lamiaceae]*	phosphorylation	↑ p-AMPK	In a mice model of hepatic fibrosis induced by CCl_4_ alone or in combination with HFHC diet; Primary HSCs cells and LX-2 cells treated with TGF-β1	40 and 150 mg/kg for 4 or 8 weeks; 5, 20, 50 µM for 24 h	Tang et al. (2024)
	Ampelopsin	*Nekemias grossedentata (Hand.-Mazz.) J.Wen and Z.L.Nie [Vitaceae]*	Phosphorylation	↓ SIRT1/TGF-β1/Smad3 signaling pathway; ↓ AKT/mTOR signaling pathway	In a mice model of hepatic fibrosis induced by CCl_4_; Primary HSCs cells treated with TGF-β1	125 and 250 mg/kg for 10 weeks; 25, 50 and 100 μM for 24 h	Ma et al. (2019)
	Physalin B	*Physalis L.[Solanaceae]*	Acetylation	↓ GLI1 deacetylation	In a mice model of hepatic fibrosis induced by CCl_4_ and BDL; Primary HSCs cells and LX-2 cells treated with TGF-β1	1, 2.5 and 5 mg/kg for 4 weeks; 0.25, 0.5 and 1 μM for 24 h	Zhu X. et al. (2021)
Phenolic Compounds	Capsaicin	*Capsicum cardenasii Heiser and P.G.Sm.* and *Capsicum L. [Solanaceae]*	Phosphorylation	↑ PPAR-γ; ↓ TGF-β1/Smad Pathway	In a rat model of hepatic fibrosis induced by DMN; HSC-T6 cells treated with TGF-β1	0.5 and 1.0 mg/kg for 4 weeks; 0.1, 1 and 10 μM for 24 h	Choi et al. (2017)
	Ferulic acid	*Angelica sinensis (Oliv.) Diels [Apiaceae]*	Phosphorylation	↓ p-Smad2; ↓ p-Smad3	In a rat model of hepatic fibrosis induced by CCl_4_; LX-2 cells treated with TGF-β1	10 mg/kg/day for 8 weeks; 50 μM for 24 h	Mu et al. (2018)
	Honokiol	*Magnolia officinalis Rehder and E.H.Wilson [Magnoliaceae]*	Phosphorylation	↑ E-cadherin/GSK3β/JNK signaling pathway; ↓ AKT/ERK/p38/β-catenin/TMPRSS4 signaling pathway	In a mice model of hepatic fibrosis induced by CCl_4_; AML-2 cells treated with TGF-β1	1 mg/kg for 6 weeks; 12, 24, 36, and 48 μM for 24 h	Seo et al. (2023)
	Epigallocatechin (ECG), Epicatechin-3-O-gallate (EGC) and Epigallocatechin-3-O-gallate (EGCG)	*Camellia sinensis (L.) Kuntze [Theaceae]*	Phosphorylation	↓ p-ERK; ↓ p-Smad1/2	In a rat model of hepatic fibrosis induced by CCl_4_	ECG (100 and 300 mg/kg), EGC (100 and 300 mg/kg),EGCG (300 mg/kg)	Wang et al. (2019)
	Astragalus and Salvia miltiorrhiza extract	*Astragalus L. [Fabaceae] and Salvia miltiorrhiza Bunge [Lamiaceae]*	Phosphorylation	↓ p-ERK, p-JNK p-P38; ↓ p-Smad2C/L, p-Smad3L, Smad4, Imp7/8	In a rat model of hepatic fibrosis induced by DEN; Primary HSCs cells treated with TGF-β1 and HepG2 cells	60,120 and 240 mg/kg for 12 or 16 weeks; 20, 40 and 80 mg/mL	Boye et al. (2015)
	Danshensu	*Salvia miltiorrhiza Bunge [Lamiaceae]*	Phosphorylation	↓ p-STAT3; ↓ JAK2-STAT3 signaling pathway	In a rat model of hepatic fibrosis induced by CCl_4_; HSC-T6 cells treated with TGF-β1	10, 30 and 60 mg/kg for 6 weeks; 1, 2, and 3 μM for 12, 24 and 48 h	Cao et al. (2019)
	Salvianolic acid B	*Salvia miltiorrhiza Bunge [Lamiaceae]*	Phosphorylation	↓ p-Smad2/3L, ↓ p-Smad2C, ↑ p-Smad3C; ↓ MAPK	In a mice model of hepatic fibrosis induced by DEN; HSC-T6 cells and LX-2 cells treated with TGF-β1	15 and 30 mg/kg for 12 weeks; 20, 50 and 100 μM for 24 h	Wu et al. (2019)
			Methylation	↓ DNMT1, ↑ PTCH1	In a mice model of hepatic fibrosis induced by CCl_4;_ Primary HSCs cells treated with TGF-β1	100 mg/kg for 8 weeks; 100 μM for 48 h	Yu et al. (2015)
	Oroxylin A	*Scutellaria baicalensis Georgi [Lamiaceae]*	Methylation	↑ cGAS and STING, ↓ cGAS gene methylation; ↓ DNMT3A	In a mice model of hepatic fibrosis induced by CCl_4_; HSC-T6 cells and LX-2 cells treated with TGF-β1	40 mg/kg for 8 weeks; 20, 30 and 40 μM for 24 h	Zhao et al. (2023)
Terpenoids	Total astragalus saponins (AST); Glycyrrhizic acid (GA)	*Astragalus mongholicus Bunge [Fabaceae]; Glycyrrhiza uralensis Fisch. ex DC. [Fabaceae]*	Phosphorylation	↓ p-Smad2/3 and TGF-β1 pathway	In a rat model of hepatic fibrosis induced by DEN and Bile duct ligation; JS-1 cells and AML-12 cells	AST (164 mg/kg)+GA (48 mg/kg), AST (164 mg/kg), GA (48 mg/kg) for 2 or 3 weeks; AST (10、20 and 40 μg/mL), GA (25、50 and 100 μM), AST: 20 μg/mL + GA: 50 μM	Zhou et al. (2016)
	Corosolic acid	*Crataegus pinnatifida* var. *pinnatifida [Rosaceae]*	Phosphorylation	↓ TGF-β1/Smad2, NF-κB, and AMPK signaling pathways	In a mice model of hepatic fibrosis induced by HFD diet and CCl4; LX-2 cells treated TGF-β1 and HepG2 cells	10, 20 and 30 mg/kg for 9 weeks; 5, 10 and 20 μM for 24 h	Liu et al. (2021)
	Cryptotanshinone	*Salvia miltiorrhiza Bunge [Lamiaceae]*	Phosphorylation	↓ p-STAT3; ↓ CTP1A fatty acid metabolism	In a mice model of hepatic fibrosis induced by CCl_4_; Primary HSCs cells and LX-2 cells treated TGF-β	40 mg/kg for 5 weeks; 1, 5 and 10 μM for 48 h	Li et al. (2024)
	Asiatic acid	*Centella asiatica (L.) Urb. [Apiaceae]*	Phosphorylation	↓ NF-κB/IκBα and JAK1/STAT3 signaling pathway	In a rat model of hepatic fibrosis induced by CCl_4_	5 and 15 mg/kg for 6 weeks	Fan et al. (2018)
	Demethylzeylasteral	*Tripterygium wilfordii Hook.f. [Celastraceae]*	Phosphorylation	↓ AGAP2, ↓ p-FAK, ↓ p-AKT	In a rat model of hepatic fibrosis induced by CCl_4_; Primary HSCs, LX-2 and HSC-T6 cells treated with TGF-β1	20 mg/kg for 4 weeks; 0.5, 1 and 2 μM for 24 h	Chen et al. (2022)
	Triptolide	*Tripterygium wilfordii Hook.f. [Celastraceae]*	Phosphorylation	↑ p-AMPK, ↑ p-ACC1 lipid metabolism	In a mice were fed a methionine/choline-supplied (MCS) or MCD diet	50 μg/kg and 100 μg/kg for 10 weeks	Huang et al. (2021)
	Celastrol	*Tripterygium wilfordii Hook.f. [Celastraceae]*	Phosphorylation	↑ p-AMPK, ↑ p-SIRT3	In a rat model of hepatic fibrosis induced by CCl_4_; Primary HSCs cells treated with PDGR-BB	0.25, 0.5 and 1 mg/kg for 8 weeks; 10, 20 and 40 μM for 24 h	Wang et al. (2020a)
	Saponin extract of *P. japonicus* rhizomes	*Panax japonicus (T.Nees) C.A.Mey. [Araliaceae]*	Phosphorylation	↑ p-Akt, ↑ p-GSK3β; ↑ Akt/GSK3β/Nrf2 cascade	In a mice model of hepatic fibrosis induced by CCl_4_; AML-12 cells treated with TGF-β	50 and 100 mg/kg for 4 weeks; 30 and 100 μg/mL for 24 h	Dai et al. (2021)
	Carnosol	*Salvia rosmarinus Spenn. [Lamiaceae]*	Acetylation	↑ SIRT1; ↓ EZH2 acetylation	In a rat model of hepatic fibrosis induced by CCl_4_; Primary HSCs cells and LX-2 cells treated with TGF-β1	25 and 30 mg/kg for 4 weeks; 10 μM for 24 h	Zhao et al. (2018)
	Sclareol	*Salvia sclarea L. [Lamiaceae]*	Ubiquitylation	↓ SENP1, ↑ VEGFR2 SUMOylation, ↓ VEGFR2–STAT3 interaction, ↓ p-STAT3	In a rat model of hepatic fibrosis induced by CCl_4_ and Bile duct ligation; LX-2 cells treated TGF-β1	300 mg/kg for 4 weeks; 10 and 20 μM for 24 h	Ge et al. (2023)
	Ginsenoside Rg1	*Panax ginseng C.A.Mey. [Araliaceae]*	Methylation	↓ DNMT1-mediated Smad7 methylation	In a mice model of hepatic fibrosis induced by CCl_4_; Primary HSCs treated TGF-β	40 mg/kg for 8 weeks, 50 μM for 24 h	Zhang et al. (2023)
	Ginsenoside Rg3	*Panax ginseng C.A.Mey. [Araliaceae]*	Methylation	↑ ACSL4, ↓ DNMT3B-mediated ACSL4 methylation	In a mice model of hepatic fibrosis induced by CCl_4_; Primary HSCs cells treated with TGF-β1	10 and 20 mg/kg for 8 weeks; 20 and 40 μM for 24 h	Hu Y. et al. (2024)
Alkaloids	Matrine (MT) Thio derivative of MT (MD-1)	*Sophora flavescens Aiton [Fabaceae]*	Phosphorylation	↓ p-EGFR and p-AKT	In a rat model of hepatic fibrosis induced by DMN; HSC-T6 cells treated with TGF-β1	MT and MD-1 (62 μmol/L/kg) for 4 weeks; MD-1 (62 µmol/L), MT (128 µmol/L) for 24 h	Feng et al. (2016)
	Berberine	*Coptis chinensis Franch. [Ranunculaceae]is*	Phosphorylation	↑ p-AMPK, ↓ p-Akt, ↓ Nox4, ↓ TGF-β1	In a mice model of hepatic fibrosis induced by CCl_4_; CFSC-2G cells	25 and 50 mg/kg for 4 weeks; 12.5 μM for 24 h	Li et al. (2014)
	Neferine	*Nelumbo nucifera Gaertn. [Nelumbonaceae]*	Phosphorylation	↑ p-AMPK and p-ACC; ↑ p-Smad2/3, TGF-β	In a mice model of hepatic fibrosis induced by HFD diet and CCl_4_; LX-2 cells treated with TGF-β and HepG2 cells	5 and 10 mg/kg for 4 weeks; 12.5 and 25 μM for 24 h	Wang M. Y. et al. (2023)
	Piperine	*Piper nigrum L. [Piperaceae]*	Phosphorylation	↑ Nrf2, ↓ p-Smad2/3, ↑ Smad7	In a mice model of hepatic fibrosis induced by CCl_4_; AML-12 cells and LX-2 cells treated with TGF-β	20 and 40 mg/kg for 4 weeks; 20 and 40 μM for 48 h	Shu G. et al. (2021)
Other Natural Drugs	Ganoderma lucidum polysaccharide	*Rhinacanthus nasutus (L.) Kurz [Acanthaceae]*	Phosphorylation	↓ TGF-β, p-Smad2 and p-Smad3	In a mice model of hepatic fibrosis induced by CCl_4_; HSC-T6 cells treated with TGF-β1	150 and 300 mg/kg for 6 weeks; 1.25, 2.5 and 5 mg/mL for 24 h	Chen C. et al. (2023)
	*S. glauca* extract (SGE)	*Suaeda glauca (Bunge) Bunge [Amaranthaceae]*	Phosphorylation	↓ p-Smad2/3 and Smad2/3 nuclear translocation	In a mice model of hepatic fibrosis induced by CCl_4_; Primary HSCs cells and LX-2 cells treated TGF-β	30 and 100 mg/kg for 6 weeks; 100 and 300 μg/mL for 30 min, 72 h or 5 days	Hong et al. (2023)
	Amygdalin	*Prunus armeniaca L. [Rosaceae]]*	Phosphorylation	↓ p-Smad2 and p-Smad3; ↓ p-p65 (NF-κB)	In a rat model of hepatic fibrosis induced by CCl_4_; LX-2 cells treated with TGF-β1	3 mg/kg for 8 weeks; 1.25, 2.5 and 5 mg/mL for 48 h	Wang et al. (2021)
	Cordycepin	*Cordyceps militaris (L.ex Fr.) Link.[clavicipitaceae]*	Phosphorylation	↑ p-AMPKα and p-ACC	In a mice model of hepatic fibrosis induced by HFHC-fed; L02 cells	100 and 200 mg/kg for 16 weeks; 50 μM for 12 h	Lan et al. (2021)
	Plumbagin	*Plumbago zeylanica L. [Plumbaginaceae]*	Phosphorylation	↓ p-IκB and NF-κB p65 nuclear translocation	In a rat model of hepatic fibrosis induced by CCl_4_; Primary HSCs cells	2, 4 and 8 mg/kg for 4 weeks; 2, 4 and 8 μM for 24 h	Chen et al. (2019)
	Inchin-ko-to	*Artemisia capillaris Thunb. [Asteraceae]; Gardenia jasminoides J.Ellis [Rubiaceae]* and *Rheum rhabarbarum L. [Polygonaceae]*	Phosphorylation	↓ phosphorylation of PDGFRβ, c-Raf, MEK1/2, ERK1/2 and Akt	In a rat model of hepatic fibrosis induced by TAA; Primary HSCs cells treated with PDGF-BB	1 mg/g for 6 weeks; 10, 50 and 100 μg/mL for 24 h or 48 h	Imanishi et al. (2004)
	Emodin	*Rheum officinale Baill. [Polygonaceae]*	Methylation	↓ p-ERK, ↓ p-Nur77, ↓ Nur77/DNMT3b interaction, ↓ GLS1 promoter methylation	In a mice model of hepatic fibrosis induced by CCl_4_; HSC-T6 cells and LX-2 cells treated with TGF-β1	30 mg/kg for 4 weeks; 10, 20 and 40 μM, or a single concentration of 20 μM for 24 h	Chen L. et al. (2023)
	Sennoside A	*Rheum officinale Baill. [Polygonaceae]*	Methylation	↑ SOCS1, ↓ DNMT1	In a rat model of hepatic fibrosis induced by CCl_4_; HSC-T6 cells treated with TGF-β1	30 mg/kg for 8 weeks; 20 nM for 24 h	Zhu H. et al. (2021)

### 4.1 Flavonoids

Flavonoids are widely distributed plant-derived compounds with multiple pharmacological properties, such as anti-inflammatory, antioxidant, anti-apoptotic, and lipid-regulating effects (Li et al., 2016; Pourcel et al., 2007; Serafini et al., 2010). They have been shown to ameliorate liver diseases, including acute liver injury and fatty liver (Blankson et al., 2000; Luo S. et al., 2023).

Recent studies demonstrate that flavonoids exert antifibrotic effects primarily through the regulation of PTMs, especially phosphorylation. For example, total flavonoids from *Scabiosa comosa* (TF-SC) selectively inhibit TGF-β1–induced Smad3 phosphorylation by blocking the TβRI–Smad3 interaction, thereby attenuating CCl_4_-induced liver fibrosis and identifying TF-SC as a potential TGF-β1/Smad3 pathway inhibitor (Ma et al., 2018). Similarly, luteolin promotes HSC apoptosis by activating caspase-3 and upregulating p53, while downregulating bcl-2, cyclin E, and p-Cdk2. *In vivo*, luteolin alleviates fibrotic injury by suppressing PDGF- and TGF-β1-mediated AKT and Smad phosphorylation (Li et al., 2015). Naringin inhibits PI3K/AKT signaling, decreases fibronectin and TGF-β1 expression, and induces caspase-3–dependent apoptosis to mitigate fibrosis (El-Mihi et al., 2017). Myricetin suppresses TGF-β1–induced phosphorylation of Smad2, p38 MAPK, ERK, and AKT, and dose-dependently inhibits PDGF-BB–induced ERK/AKT activation. In CCl_4_ models, myricetin reduces α-SMA expression and collagen deposition, indicating multitarget regulation of PTMs (Geng et al., 2017). Quercetin attenuates HSC activation by downregulating p38 MAPK phosphorylation and modulating the NF-κB/IκBα axis (Wang et al., 2017).

Other flavonoids demonstrate similar PTM-mediated mechanisms. Limonin blocks TGF-β–induced Smad2/3 phosphorylation and nuclear translocation, while enhancing Smad7 expression to suppress epithelial–mesenchymal transition (EMT) and HSC activation (Shu et al., 2022). Isoliquiritigenin inhibits STAT3 phosphorylation by targeting ANXA2 and the SPHK/S1P/IL-17 pathway, reversing HSC activation (Liu et al., 2023). Catechin-7-O-β-d-apiofuranoside (C7A) represses TGF-β1–induced STAT3 phosphorylation and downstream ECM gene expression, reducing fibrosis (Park et al., 2019). In addition, luteolin-7-diglucuronide (L7DG) has been identified as an inhibitor of protein tyrosine phosphatase 1B (PTP1B), which promotes AMPK phosphorylation and suppresses cell activation in TGF-β1–stimulated HSCs (Tang et al., 2024).

Beyond phosphorylation, other PTMs also play important roles in the anti-fibrotic mechanisms of flavonoids. Ampelopsin (Amp) has been shown to exert regulatory effects largely through deacetylation. Specifically, Amp downregulates collagen I, α-SMA, TIMP1, TGF-β1, and p-Smad3 expression, while promoting the upregulation of MMP9 and SIRT1, thereby inhibiting the sustained activation of HSCs. Importantly, SIRT1 activation is central to this process, as its inhibition reverses the protective effects of Amp. Moreover, Amp induces autophagy by upregulating LC3-II and Beclin-1, whereas the autophagy inhibitor 3-MA partially abrogates its anti-fibrotic effects (Ma et al., 2019). Physalin B enhances the acetylation of the transcription factor GLI1, preventing its interaction with the LAP2α/HDAC1 complex, leading to its inactivation, downregulation of α-SMA and COL1A1 expression, and ultimately exerting anti-fibrotic activity (Zhu X. et al., 2021).

In summary, flavonoids and related bioactive compounds exert anti-fibrotic effects not only through classical signaling pathways such as TGF-β/Smad, PI3K/AKT, MAPK, STAT3, and NF-κB, but more importantly through the regulation of diverse PTMs. Phosphorylation is central to blocking the Smad, AKT, and MAPK axes; deacetylation and acetylation regulate HSC fate via SIRT1 activation and GLI1 inactivation, respectively; and autophagy induction contributes to the suppression of fibrosis progression. These findings highlight PTMs as critical therapeutic targets of natural compounds, underscoring their multi-target and multi-pathway advantages and providing a solid theoretical foundation for the development of novel PTM-oriented anti-fibrotic drugs.

### 4.2 Phenolic compounds

Phenolic compounds are naturally occurring substances abundant in fruits, vegetables, grains, legumes, chocolate, and beverages such as tea and wine (Spencer et al., 2008). They display diverse pharmacological properties, including anti-inflammatory, antioxidant, anti-proliferative, lipid-regulating, and anti-aging activities (Toma et al., 2020; Urso and Clarkson, 2003; Van Hung, 2016). Clinically, phenolics have been applied in the management of hypertension, metabolic disorders, infections, and neurodegenerative diseases (Lin et al., 2016; Rahman et al., 2021). Increasing evidence supports their therapeutic role in HF through the regulation of PTMs and epigenetic processes.

Capsaicin (CPS) has been shown to upregulate Smad7 expression through the activation of PPAR-γ, thereby suppressing DMN-induced TGF-β1 production. In HSCs, CPS effectively reduced TGF-β1–mediated α-SMA and collagen I expression via this pathway, suggesting that it ameliorates fibrosis by negatively regulating the TGF-β1/Smad signaling axis through the PPAR-γ/Smad7 pathway (Choi et al., 2017). Similarly, ferulic acid (FA) markedly inhibits the phosphorylation of Smad2/3 and the downstream signal transduction of Smad4, thereby attenuating TGF-β1–induced HSC activation and contributing to the reversal of fibrosis progression (Mu et al., 2018). Honokiol exhibits broader multi-pathway regulatory effects. In HSCs, honokiol reduces the expression of α-SMA, TGF-β1, p-Smad3, p-AKT, Cyclin D1, c-Myc, and Wnt3a/β-catenin, while inhibiting the phosphorylation of GSK3β, which leads to GSK3β activation, blockade of Wnt3a/β-catenin signaling, and apoptosis induction (Lee et al., 2021). Further studies revealed that honokiol also suppresses non-canonical TGF-β1 pathways (AKT, ERK, and p38) while promoting GSK3β/JNK phosphorylation, which enhances E-cadherin expression and inhibits EMT progression. *In vivo*, honokiol has been shown to attenuate CCl_4_-induced hepatic fibrosis and necrosis (Seo et al., 2023).

Tea polyphenols also confer hepatoprotection. Green tea catechins [epigallocatechin (EGC), epicatechin-3-O-gallate (ECG), and epigallocatechin-3-O-gallate (EGCG)] significantly reduce desmin, α-SMA, and TGF-β expression, while inhibiting the phosphorylation of ERK1/2 and Smad1/2, thereby ameliorating fibrosis (Wang et al., 2019). Sugarcane polyphenol extract (SPE) has also been shown to inhibit the phosphorylation of p38 and JNK1/2 and downregulate α-SMA expression in TGF-β1–induced HSCs (Wang L. et al., 2018). In addition, the compound extract of *Astragalus and Salvia miltiorrhiza* (CASE) suppresses linker-region phosphorylation of Smad2/3 and the nuclear import of Smad4 and Imp7/8, thereby reducing the transcriptional activity of PAI-1, a key target gene of TGF-β signaling. This effect is accompanied by inhibition of the MAPK pathway (pERK, pJNK), ultimately suppressing the fibrotic response of HSCs (Boye et al., 2015).

Among active compounds from *S. miltiorrhiza*, Danshensu (DSS) is identified as an inhibitor of indoleamine 2,3-dioxygenase 1 (IDO1). DSS downregulates JAK2/STAT3 signaling by reducing JAK2/STAT3 phosphorylation and STAT3 nuclear localization, thereby inhibiting ECM deposition and liver injury. Overexpression of IDO1 reverses these effects, confirming its role in fibrosis (Cao et al., 2019). Salvianolic acid B (Sal B) suppresses fibrosis via the MAPK/Smad axis and Hedgehog pathway, significantly reducing p-ERK1/2, p-JNK1/2, p-p38, p-Smad2/3, and PAI-1 levels (Wu et al., 2019). In addition, Sal B attenuates HSC activation by regulating miR-152/DNMT1-mediated DNA methylation, thereby suppressing the hypermethylation of PTCH1 and restoring its expression (Yu et al., 2015). Oroxylin A further demonstrates epigenetic action by inhibiting DNMT3A-mediated methylation of the cGAS promoter. This activates the cGAS–STING pathway and induces cellular senescence, while DNMT3A overexpression reverses these effects (Zhao et al., 2023).

In summary, phenolic compounds—including alkaloids, phenolic acids, polyphenols, and herbal extracts—exert potent antifibrotic effects by targeting both canonical and noncanonical pathways (TGF-β/Smad, MAPK, PI3K/AKT, Wnt/β-catenin, and JAK/STAT), as well as by modulating DNA methylation and other epigenetic processes. Their ability to act at multiple levels of regulation underscores PTMs and epigenetic remodeling as promising therapeutic intervention points, providing a strong foundation for the development of novel antifibrotic strategies.

### 4.3 Terpenoids

Terpenoids are a large and structurally diverse class of bioactive natural compounds found in plants, animals, marine organisms, and microorganisms. They exhibit a wide spectrum of pharmacological properties, including antitumor cytotoxicity, neuroprotection, anti-inflammatory activity, regulation of lipid metabolism, as well as hepatoprotective and hypoglycemic effects (Cassiano et al., 2014; El Omari et al., 2021; Hua et al., 2022; Yao and Liu, 2022).

Co-administration of total astragalosides (AST) and glycyrrhizic acid (GA) markedly inhibits HSC activation, lowers α-SMA and COL1A1 expression, and suppresses transcription and phosphorylation of TGF-β1 and Smad2/3, thereby reversing dimethylnitrosamine (DMN)– or bile duct ligation (BDL)–induced hepatic fibrosis (Zhou et al., 2016). Similarly, corosolic acid (CA) mitigates fibrosis by blocking TGF-β1/Smad2 phosphorylation and concomitantly regulating the AMPK and NF-κB pathways, which decreases ECM deposition and inflammation (Liu et al., 2021). Ginsenoside Rg1 restores Smad7 expression through promoter demethylation, blocking TGF-β/Smad signaling and suppressing epithelial–mesenchymal transition (EMT); consistent with this mechanism, the DNMT inhibitor 5-Aza also enhances Smad7 demethylation and expression (Zhang et al., 2023).

Several terpenoids act through STAT3 modulation. Cryptotanshinone (CTS), a derivative of tanshinone IIA, inhibits STAT3 nuclear translocation, reduces CPT1A-dependent fatty acid oxidation, and promotes an adipocyte-like phenotype in HSCs, collectively producing antifibrotic effects. In CCl4-induced fibrosis, p-STAT3 co-localizes with the HSC activation marker α-SMA; CTS reduces p-STAT3 and favors HSC adipogenic reprogramming, thereby attenuating fibrosis (Li et al., 2024). Similarly, Asiatic acid (AA) further suppresses JAK1/STAT3 phosphorylation, preventing persistent pathway activation and ameliorating fibrosis (Fan et al., 2018). Sclareol (SCL) suppresses SENP1 expression, augments VEGFR2 SUMOylation, and disrupts the VEGFR2–STAT3 interaction, thereby inhibiting downstream STAT3 phosphorylation and providing new evidence that SUMOylation contributes to antifibrotic effects (Ge et al., 2023).

Other terpenoids target cytoskeletal and metabolic signaling. Demethylzeylasteral (T-96), from *Tripterygium wilfordii*, selectively inhibits FAK and AKT phosphorylation and disrupts the AGAP2–FAK interaction, suppressing HSC proliferation and migration (Chen et al., 2022). Triptolide acts as an AMPK agonist, increases AMPK Thr172 phosphorylation and phosphorylation of its downstream substrate ACC1, thereby attenuating fibrosis (Huang et al., 2021). Similarly, celastrol mitigates hepatic fibrosis by activating the AMPK–SIRT3 axis, improving mitochondrial homeostasis and anti-inflammatory defenses (Wang et al., 2020a). Likewise, the ginsenoside metabolite 20(S)-protopanaxadiol (20S-PPD) activates LKB1-dependent AMPK Thr172 phosphorylation, downregulates the mTOR/S6K pathway, and promotes HSC apptosis (Park et al., 2017).

Other mechanisms have also been reported. The saponin extract of *Panax japonicus* rhizomes (SEPJ) augments phosphorylation of AKT and GSK-3β to activate NRF2 signaling, upregulates NRF2 and its downstream antioxidant genes, thereby inhibiting EMT and HSC activation, and demonstrates antifibrotic efficacy *in vitro* and *in vivo* (Dai et al., 2021). Carnosol (CS) activates SIRT1 to reduce EZH2 acetylation and stability, limiting myofibroblast differentiation and ECM accumulation (Zhao et al., 2018). Ginsenoside Rg3 augments ferroptosis through ACSL4 demethylation to restrain HSC activation; this effect is mediated via the miR-6945-3p/DNMT3B axis, unveiling a ferroptosis-based mechanism underlying Rg3’s antifibrotic activity (Hu Y. et al., 2024).

### 4.4 Alkaloids

Alkaloids are nitrogen-containing basic compounds with broad pharmacological activity. More than 18,000 alkaloids have been identified across >300 plant families as well as from microorganisms, marine invertebrates, insects, and other sources (Elissawy et al., 2021; Nair and van Staden, 2019). Research has demonstrated that various alkaloids possess pharmacological effects such as antihypertensive, anti-inflammatory, anticancer, and anti-fibrotic properties (Bhambhani et al., 2021; Feng et al., 2016; Halim et al., 2019).

Mechanistically, representative alkaloids modulate key PTM-linked signaling nodes to restrain HSC activation and ECM accumulation. The matrine derivative MD-1 engages EGFR on HSC-T6 cells to suppress EGFR/AKT phosphorylation, downregulate cyclin D1, and block persistent HSC activation; in DMN–induced rat fibrosis, MD-1 attenuates disease progression, improves liver function, and preserves hepatocyte integrity (Feng et al., 2016). Berberine (BBR) exerts antifibrotic activity predominantly via AMPK activation with concomitant repression of NOX4/AKT expression, thereby reducing oxidative stress and ECM deposition (Li et al., 2014). Neferine (NEF) protects against NASH-associated fibrosis by inhibiting TGF-β/Smad2/3 signaling, preventing HSC activation and downregulating profibrotic genes (Wang M. Y. et al., 2023). In AML-12 hepatocytes and LX-2 HSCs, piperine (PIP) drives NRF2 nuclear translocation and antioxidant gene transcription to limit TGF-β1–elicited ROS; concurrently it increases SMAD7 while restraining SMAD2/3 phosphorylation/nuclear entry, an effect blunted by NRF2 knockdown, implicating NRF2 as a key mediator (Shu G. et al., 2021).

Collectively, these alkaloids converge on receptor signaling (EGFR), metabolic/oxidative pathways (AMPK/NOX4), and canonical profibrotic axes (TGF-β/Smad) with NRF2 crosstalk, highlighting PTM-centered mechanisms as tractable targets for antifibrotic intervention.

### 4.5 Other natural drugs

Accumulating evidence indicates that natural products of varied origin mitigate hepatic fibrosis by tuning PTMs across key signaling nodes. *Ganoderma lucidum* polysaccharide (GLP) lowers hepatic TGF-β and SMAD2/3 phosphorylation in CCl4-injured mice while restoring SMAD7, thereby preventing activation of HSC-T6 cells (Chen C. et al., 2023). Likewise, *Suaeda glauca* extract (SGE) suppresses TGF-β1–evoked SMAD2/3 phosphorylation and nuclear translocation and reduces SBE-dependent transcriptional activity—without affecting JNK or ERK—implicating selective blockade of the TGF-β/SMAD axis (Hong et al., 2023). Moreover, amygdalin diminishes TGF-β/SMAD2/3 phosphorylation and downregulates profibrotic gene expression, thereby curbing HSC activation and ECM deposition and mitigating CCl4-induced hepatic injury (Wang et al., 2021).

Several agents act through coordinated pathway inhibition. In HSCs, emodin reduces TGF-β1, TβRI/TβRII, and SMAD4 expression and markedly suppresses SMAD-responsive luciferase activity and p38 MAPK activation. Notably, single inhibition of SMAD4 or p38 only partially attenuates emodin’s effect, whereas combined inhibition abolishes it, indicating that emodin represses collagen gene transcription via cooperative blockade of SMAD and p38 MAPK signaling (Wang X. et al., 2018). Chrysophanol 8-O-glucoside (C8G) selectively inhibits p38 phosphorylation and blocks STAT3 activation/nuclear import, reducing MMP2 expression and ECM accumulation (Park et al., 2020). In NASH, cordycepin augments AMPK phosphorylation to restrain NF-κB activation and, via ACC phosphorylation, corrects lipid dysregulation, yielding anti-inflammatory and antifibrotic benefits; AMPK inhibition abrogates these effects, underscoring AMPK dependence (Lan et al., 2021). Plumbagin (PL) lowers IκB phosphorylation in CCl4-injured rat liver and IL-1β–stimulated HSCs, preventing NF-κB nuclear entry/transactivation and thus mitigating inflammation and ECM build-up (Chen et al., 2019). The Kampo formula Inchin-ko-to (TJ-135) decreases collagen deposition and α-SMA in fibrotic rats and represses COL1A1, COL3A1, and fibronectin transcription in HSCs by inhibiting PDGFRβ phosphorylation and downstream signaling (Imanishi et al., 2004).

Epigenetic targeting is also prominent. Emodin suppresses ERK/Nur77 signaling, drives Nur77 nuclear translocation and DNMT3b binding, increases GLS1 promoter methylation, inhibits glutaminolysis, triggers energetic stress and HSC senescence, and thereby exerts antifibrotic activity (Chen L. et al., 2023). Sennoside A (SA) upregulates SOCS1 in a DNMT1-dependent manner and suppresses macrophage pro-inflammatory cytokines, indirectly limiting HSC proliferation and ECM deposition; SOCS1 blockade diminishes efficacy, highlighting a pivotal epigenetic component in SA’s antifibrotic action (Zhu H. et al., 2021).

These natural products—polysaccharides, halophyte extracts, anthraquinones, nucleosides, naphthoquinones, and multi-component formulas—converge on PTM-regulated nodes (e.g., SMAD, p38, STAT3, AMPK/ACC, NF-κB, PDGFRβ) and epigenetic machinery (DNMT1/DNMT3b, promoter methylation) to suppress HSC activation, inflammation, and ECM deposition. Their multi-target profiles reinforce PTMs and epigenetic remodeling as tractable intervention points for antifibrotic drug development.

## 5 The prospects and challenges of traditional medicine in the treatment of liver fibrosis

Natural products have long served as a vital source for drug discovery because of their broad biological activities. Effective antifibrotic constituents are generally categorized into flavonoids, saponins, alkaloids, and other classes (Liu et al., 2022; Liu et al., 2020; Sharma et al., 2024; Zhao et al., 2023a). Their antifibrotic mechanisms include inhibition of hepatic inflammation, suppression of lipid-peroxidation injury, regulation of the synthesis and secretion of profibrotic factors, modulation of ECM synthesis and degradation, and inhibition of HSCs activation and proliferation. These effects are tightly linked to the regulation of PTMs, and this multi-target strategy offers distinct advantages for addressing the complexity of liver fibrosis (Li et al., 2023; Ma et al., 2020).

A growing body of *in vivo* and *in vitro* evidence indicates that natural products can attenuate fibrosis by tuning PTMs (phosphorylation, acetylation, methylation, SUMOylation). However, current PTM research is heavily skewed toward phosphorylation, whereas other PTMs remain sparsely studied. This imbalance reflects not only biological interest but also methodological convenience: phosphoproteomic enrichment and mass-spectrometry workflows are mature and widely accessible, whereas systematic profiling of lysine acetylation, ubiquitination, or emerging PTMs such as succinylation or malonylation requires higher cost, more complex sample preparation, and specialized antibodies or chemical probes. As a result, kinase/phosphatase axes dominate the mechanistic landscape, potentially obscuring druggable “writers,” “erasers,” and “readers” of other PTMs and underestimating crosstalk among PTM layers. Such bias limits discovery of alternative regulatory nodes in HSC activation and ECM dynamics and may hinder translation if therapeutic development focuses narrowly on kinase pathways.

Clinical translation remains nascent. Small randomized trials in non-alcoholic fatty liver disease (NAFLD) suggest that certain flavonoids (e.g., hesperidin) improve liver and metabolic indices (El-Mihi et al., 2017). Human studies of the green-tea catechin EGCG report heterogeneous effects on steatosis and inflammation, and dose-related hepatotoxicity has been documented (Wang et al., 2019; Yu et al., 2015). Components of Salvia miltiorrhiza (e.g., salvianolic acid B, danshensu) and the alkaloid berberine show partial clinical benefit in metabolic or cardiovascular settings, yet evidence specific to liver fibrosis remains largely preclinical or exploratory (Wu et al., 2019; Li et al., 2014). From an evidence-quality perspective, major limitations persist: small sample sizes, inadequate randomization or controls, and pronounced heterogeneity in dosing, administration routes, and experimental models, all of which compromise reproducibility and cross-study comparison. Variability in extraction, purity, and chemical characterization further contributes to divergent findings. Conflicting effects—such as inconsistent impacts of green-tea catechins on HSC activation and ECM deposition—raise concerns about model dependence, selective reporting, and publication bias (Spencer et al., 2008; Van Hung, 2016). Safety is equally critical: EGCG-associated hepatotoxicity underscores the need for integrated toxicology and drug–drug interaction assessments alongside clinical evaluation (Wang et al., 2019).

Pharmacokinetic and formulation barriers compound these challenges. Many flavonoids, phenolics, and saponins display poor oral bioavailability, rapid metabolism, and variable systemic exposure, making it difficult to achieve therapeutic concentrations *in vivo* (Zhang et al., 2023; Zhao et al., 2018). Nanodelivery systems, liposomal encapsulation, and prodrug strategies have improved exposure and efficacy in animal models, but scale-up manufacturing and long-term safety remain to be validated (Hu Y. et al., 2024).

### 5.1 Future directions

To overcome these limitations and correct the PTM imbalance, priority areas include: i. expanding research from single-PTM (phosphorylation) analysis to *multi-PTM network* interrogation using integrated phosphoproteomics, acetylomics, ubiquitinomics, and emerging succinyl/malonyl modifications; ii. systematically evaluating PTM “writers,” “erasers,” and “readers” (e.g., deacetylases, ubiquitin ligases, SUMO E3 ligases, methyltransferases) as potential therapeutic targets; iii. validating candidate PTM changes not only in animal and cell models but also in human liver tissues and circulating biomarkers; iv. advancing compounds with defined pharmacokinetics, acceptable safety, and concordant *in vitro*/*in vivo* mechanisms into standardized Phase I/II trials (dose finding, PK/PD, interaction studies), followed by multicenter randomized trials with long-term antifibrotic endpoints; and v. implementing rigorous standards for extraction, chemical characterization, and reporting of PTM data to reduce selective reporting and publication bias. Only by broadening the PTM perspective beyond phosphorylation, integrating high-throughput epigenomics and systems biology, and coupling mechanistic insight with early pharmacokinetic/toxicological evaluation can natural-product discoveries be efficiently translated into clinically useful antifibrotic therapeutics.
